# New Perspectives in Prevention, Control, and Treatment of Dengue Fever

**DOI:** 10.3390/v18101162

**Published:** 2026-10-19

**Authors:** Jairo Castellar-Lopez, Yeini Julieth Gutiérrez Rodríguez, Gabriela Alejandra Cáceres, Monica Gomez Pulido, Gabriela Lara Barreto, Noemi Ramirez, Santiago Alcazar Santos, Marian Mares Blancas, Diego Castro Garcia, Joseph León Insignares, Vanessa Cohen Ariza, Vanessa Gamarra Nicoliche, Wendy Rosales, Ronald Maestre-Serrano, Aileen Y. Chang, Evelyn Mendoza-Torres

**Affiliations:** 1 Faculty of Health Sciences, Universidad de la Guajira, Riohach 440002, Colombia; jacastellar@uniguajira.edu.co; 2 Faculty of Health, Exact and Natural Sciences, Universidad Libre Seccional Barranquilla, Barranquilla 081001, Colombia; yeinisj-gutierrezr@unilibre.edu.co (Y.J.G.R.); gabrielaa-caceresc@unilibre.edu.co (G.A.C.); monicae-gomezp@unilibre.edu.co (M.G.P.); gabrielal-larab@unilibre.edu.co (G.L.B.); josephd-leoni@unilibre.edu.co (J.L.I.); vanessa-cohena@unilibre.edu.co (V.C.A.); vanessaj-gamarran@unilibre.edu.co (V.G.N.); wendyd.rosalesr@unilibre.edu.co (W.R.); ronaldy.maestres@unilibre.edu.co (R.M.-S.); 3 Faculty of Medicine, Centro Universitario de Ciencias de la Salud (CUCS), Universidad de Guadalajara, Guadalajara 44350, Mexico; noemi.ramirez6152@alumnos.udg.mx (N.R.); santiagosantos215@gmail.com (S.A.S.); marmaresblancas@gmail.com (M.M.B.); emi.cstrg@gmail.com (D.C.G.); 4 Department of Medicine, Foggy Bottom Campus, George Washington University, Washington 20052, DC, USA; chang@email.gwu.edu

**Keywords:** dengue, treatment, fever, infectious disease, epidemiology

## Abstract

Dengue is a mosquito-borne viral disease primarily transmitted by *Aedes aegypti* and is endemic in tropical and subtropical regions worldwide. It represents a major global public health challenge because of its rapid expansion and increasing incidence. Clinical manifestations range from mild febrile illness to severe disease with hemorrhage and death. No specific antiviral treatment exists, and prevention has relied on vector control to reduce human–mosquito contact. Although vaccines are available, access remains limited. In the Americas, dengue transmission has intensified markedly in recent years. The largest epidemic occurred in 2024, with more than 12.6 million cases and over 7700 deaths. In 2025, reports showed 4 million suspected cases, including 8966 severe cases and 2207 deaths. This persistent burden may be driven by climate variability, urbanization, mobility, environmental change, and widespread vector distribution, all of which contribute to ongoing transmission and disease severity. These trends underscore the critical need to strengthen surveillance, improve integrated vector management, expand vaccine access, and develop effective therapies to reduce the health, social, and economic impact of this disease. Accordingly, effective preventive and therapeutic strategies are needed to address this persistent and growing threat.

## 1. Introduction

Dengue virus (DENV) is the causative agent of dengue, a viral infection transmitted by mosquitoes of the genus Aedes, primarily the species *Aedes aegypti* and *Aedes albopictus*. Dengue is endemic in tropical and subtropical regions where these vectors are present [1]. However, its distribution and incidence depend not only on the vector’s characteristics but also on environmental, social, and demographic factors, such as unplanned urbanization, climate variability, and migration from endemic areas. These factors have contributed to the spread of dengue to regions where it was not previously considered a significant public health problem [1,2].

From a clinical standpoint, dengue presents a wide spectrum of manifestations that depend on factors such as the host’s immune response, age, and the viral serotype involved. According to the World Health Organization (WHO) classification, dengue can be classified as dengue without warning signs, dengue with warning signs, and severe dengue [2]. Dengue without warning signs is the most common form and is characterized by fever, headache, myalgia, retro-orbital pain, nausea, and vomiting, among other nonspecific symptoms [3]. Dengue with warning signs includes manifestations such as persistent abdominal pain, persistent vomiting, mucosal bleeding, lethargy or irritability, and evidence of plasma leakage [3]. Severe dengue is associated with complications such as significant thrombocytopenia, hemoconcentration, shock due to plasma leakage, and severe organ involvement, reflecting increased vascular permeability and a higher risk of mortality [3,4].

Epidemiologically, dengue transmission patterns can be classified according to the circulating serotypes within a community. Dengue is considered epidemic when a single serotype predominates during a specific outbreak, while hyperendemic dengue occurs when multiple serotypes circulate simultaneously in the same region, increasing the risk of secondary infections and, consequently, severe disease. Dengue may also exhibit endemic transmission, characterized by sustained transmission over time with seasonal fluctuations, frequently influenced by climatic and ecological factors [5].

Dengue has become a major global public health problem, with a substantial increase in reported cases and geographic expansion over the past five decades. Before 1970, severe dengue epidemics were reported in only nine countries. Currently, dengue is endemic in more than 100 countries, particularly in the Americas, Southeast Asia, and the Western Pacific [2,4,6]. The WHO surveillance data indicate a marked increase in the global burden of dengue since 2000, with particularly pronounced increases in recent years [2,6,7].

In the Americas, the burden of dengue has also increased markedly over recent decades [2]. This trend became especially evident in 2023 and 2024, with 4.5 million cases reported in 2023 and more than 12.9 million cases in 2024, the largest epidemic recorded in the region to date [8]. Although approximately 4.4 million cases were reported in 2025, dengue continued to represent a substantial public health burden, with Brazil accounting for a large proportion of reported cases [9]. These recent trends highlight the continued expansion and epidemiological importance of dengue throughout the Americas [8,9].

In Colombia, for example, dengue is a high-priority public health problem, with endemic transmission in approximately 600 municipalities nationwide [10,11]. Historically, incidence has increased significantly during epidemic years; for example, in 2015, 96,444 cases and 115 deaths were reported, while in 2016, 101,016 cases and 126 deaths were reported, consistent with the epidemic behavior observed at the regional level [10,11]. Subsequently, in 2019, a major outbreak occurred, with more than 127,000 reported cases, consistent with the cyclical dynamics of dengue in the Americas, where epidemics occur every 3 to 5 years [12]. More recently, consistent with regional trends, Colombia has experienced a substantial increase in cases during 2023 and 2024, reinforcing dengue as a re-emerging disease associated with substantial morbidity and mortality in the country [13].

There is no specific antiviral treatment for this disease, which remains a major challenge given the high global incidence of dengue [2]. Therefore, clinical management is based on supportive measures aimed at alleviating symptoms, such as adequate hydration, rest, fever control, medical monitoring, and, when necessary, platelet transfusion [1,14].

Given the global impact and incidence of dengue, as well as the lack of specific treatments that act directly on the virus beyond symptomatic management, this article aims to review and analyze new therapeutic alternatives targeting DENV to help mitigate the global burden of disease, reduce the risk of infection in the population, and improve public health outcomes.

## 2. Transmission of Dengue Virus

Dengue is caused by DENV, which belongs to the Orthoflavivirus genus of the family Flaviviridae. DENV is a positive-sense RNA virus that encodes three structural proteins: C (capsid), prM/M (pre-membrane/membrane), and E (envelope), as well as seven nonstructural proteins (NS1, NS2A, NS2B, NS3, NS4A, NS4B, and NS5) involved in viral replication [5,15,16].

**Figure 1:**
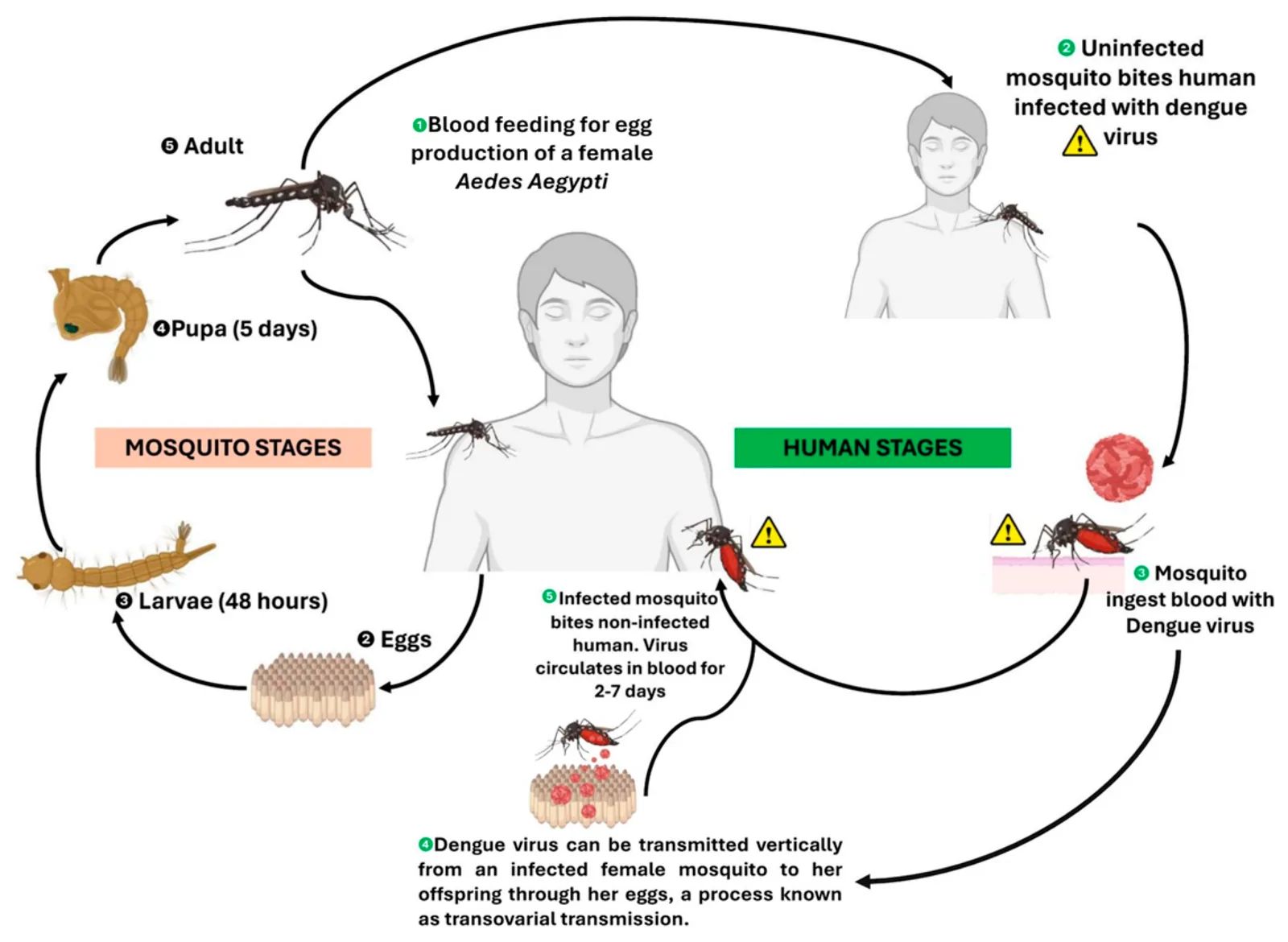
Dengue virus transmission and mosquito life cycle in *Aedes aegypti.* Mosquito stages include: (1) blood feeding taken by an adult female Aedes aegypti to support egg production, (2) egg stage, (3) larval development, (4) pupal stage, and (5) emergence of the adult mosquito. Human transmission stages include: (1) a blood feeding taken by a female *Aedes aegypti*, (2) acquisition of dengue virus when an uninfected mosquito feeds on a viremic human, (3) ingestion of virus-containing blood and subsequent viral replication and dissemination within the mosquito, (4) possible vertical transmission of dengue virus from an infected female mosquito to her offspring through the eggs (transovarial transmission), and (5) transmission of dengue virus to a susceptible human during a subsequent blood feeding, followed by viremia that can persist for approximately 2–7 days [17]. Created in BioRender. Castellar, J. (2026). https://BioRender.com/u1asb6o (accessed on 12 February 2026).

DENV is transmitted through the bites of infected female *Aedes aegypti* and *Aedes albopictus* mosquitoes, which acquire the virus when they take a blood feeding from a viremic human (Figure 1) [17]. Following ingestion of an infectious blood feeding, the virus infects and replicates in the mosquito midgut. It subsequently disseminates through the hemocoel and reaches the salivary glands. Once the virus is present in the salivary glands, the mosquito becomes capable of transmitting the virus to a susceptible host during subsequent blood meals [17]. Following transmission from an infected mosquito, DENV enters the bloodstream of the human host, initiating the intrinsic incubation period. The virus then infects susceptible target cells, including immune and endothelial cells, and uses the host cellular machinery for replication [17]. Viral replication may subsequently lead to clinical manifestations. During the viremic phase, an infected human can transmit DENV to another Aedes mosquito when the mosquito takes a blood feeding. The newly infected mosquito can then contribute to the continuation of the mosquito-human transmission cycle [17].

Each serotype is genetically distinct from the others and is subdivided into different genotypes according to phylogenetic analysis [17]. A meta-analysis published in 2020 reported that the most pathogenic serotypes in South Asia are DENV-3 and DENV-4; DENV-2 and DENV-1 are the most pathogenic in countries such as Peru, Cuba, France, Colombia, and Brazil [18].

The efficiency of human-to-mosquito DENV transmission depends strongly on the magnitude of human viremia. Direct mosquito-feeding studies have demonstrated a dose-dependent relationship between plasma viral load and the probability of Aedes aegypti infection. The estimated viral load required to infect 50% of exposed mosquitoes (MID_50_) differs among serotypes, with values of approximately 6.51 and 6.29 log_10_ RNA copies/mL for DENV-1 and DENV-2, respectively, compared with 7.52 and 7.49 log_10_ RNA copies/mL for DENV-3 and DENV-4. Human infectiousness is generally greatest during the viremic period around illness onset; however, presymptomatic and asymptomatic individuals may also infect mosquitoes and contribute to transmission. Thus, human-to-mosquito transmission depends not only on the presence of circulating virus but also on viral concentration, DENV serotype, stage of infection, and host immune responses [19,20].

Researchers have not established a universal mosquito viral-load threshold that predicts efficient DENV transmission to humans. Successful transmission requires the virus to overcome several physiological barriers within the mosquito, including midgut infection and escape barriers and salivary-gland infection and escape barriers, before it can release infectious virions in saliva. Vector competence therefore depends on mosquito species and genotype, antiviral immune responses, viral serotype and genotype, initial infectious dose, and environmental conditions, particularly temperature, which affects the extrinsic incubation period. At 25–28 °C, this period generally lasts approximately 8–12 days [21].

## 3. Epidemiology of Dengue

In the Americas, dengue has shown a sustained increase in incidence in recent years, becoming one of the arboviral diseases with the greatest impact on public health. In 2023, more than 4.5 million dengue cases were reported in the region, representing the highest annual number recorded up to that time. Of these cases, approximately 0.17% were classified as severe dengue, with a substantial burden reported in countries including Brazil, Colombia, Mexico, Peru, and Bolivia [8,9,22]. The distribution of dengue across the region remains heterogeneous, with Brazil accounting for a particularly large proportion of reported cases [8,9]. In 2024, the Americas experienced the largest dengue epidemic documented to date, with more than 12.9 million cases and more than 8000 deaths reported, demonstrating a marked intensification of transmission [2,6,8,9].

Outside the Americas, substantial dengue activity has also been reported in several Asian countries. In Bangladesh, where dengue is endemic, an unusual increase in cases was observed during 2023. Between 1 January and 7 August, the Ministry of Health and Family Welfare reported 69,483 dengue cases and 327 associated deaths, corresponding to a case fatality rate of approximately 0.47% [2,6].

During the same period, Nepal also experienced a major dengue outbreak affecting seven provinces. Although the circulating serotypes were not reported, the outbreak represented one of the largest recorded in the country based on the number of reported cases compared with previous annual totals [2,6].

## 4. Clinical Classification and Current Management of Dengue

**Figure 2:**
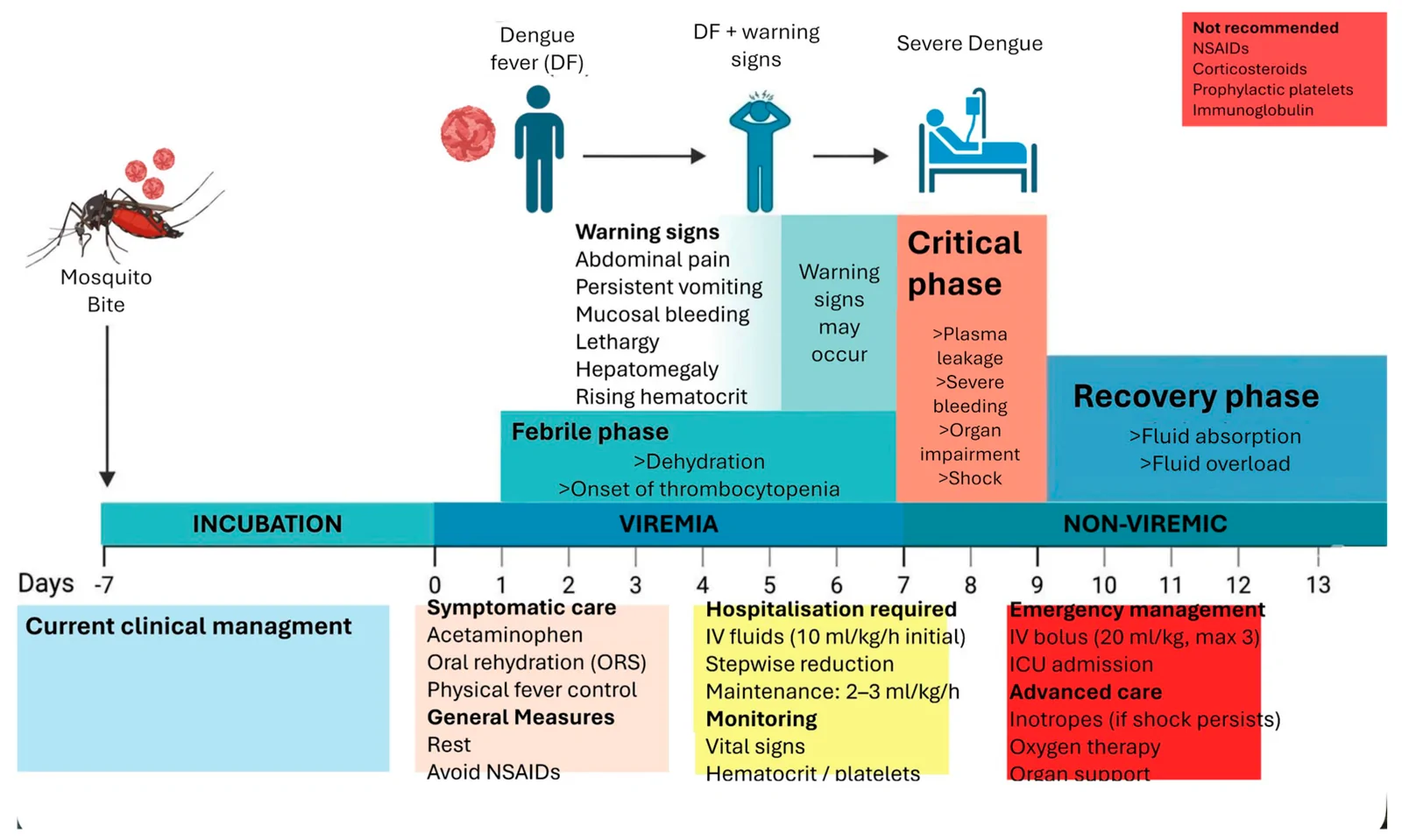
Clinical course and management of dengue infection. Created in BioRender. Castellar, J. (2026) https://BioRender.com/u1asb6o (accessed on 12 February 2026).

According to the 2025 WHO guidelines (Figure 2), management of patients with dengue without warning signs begins with supportive measures for symptom control, including antipyretics such as paracetamol for fever and general malaise while avoiding the use of nonsteroidal anti-inflammatory drugs (NSAIDs) because of the risk of hemorrhagic complications [23]. If oral intake is tolerated, increased fluid intake using standardized oral rehydration protocols is a fundamental component of care during the febrile phase. In addition, patients should be educated about warning signs and the need for clinical follow-up. Follow-up should be individualized according to disease progression, and patients should be instructed to seek immediate medical attention if signs of progression to severe disease appear [23].

After the febrile phase, which generally lasts 3 to 4 days, the critical phase begins [23,24]. At this stage, healthcare providers should closely monitor patients because the appearance of warning signs may indicate progression to more severe disease. It is important to determine whether the patient belongs to a high-risk group, including pregnant women, older adults, patients with uncontrolled diabetes mellitus, those with heart disease or chronic kidney disease, and children younger than 5 years [23]. If the patient belongs to this group, hospitalization is necessary, and fluid management should be initiated intravenously or orally, depending on tolerance [24]. The cornerstone of treatment is appropriate fluid management to preserve hemodynamic stability and tissue perfusion [24]. However, overhydration should be avoided because fluid overload can cause pulmonary edema, pleural effusion, and ascites [24]. For these patients, when warning signs are present, intravenous fluids at 10 mL/kg/h are recommended for the first 2 h; the rate should then be gradually reduced according to the patient’s response to a maintenance rate of 2–3 mL/kg/h [24].

Patients with signs of severe hypotension, shock, significant hemorrhage, respiratory distress, or multiple organ failure should be urgently admitted to an intensive care unit [25]. Intravenous fluids should be administered, preferably Ringer’s lactate at 20 mL/kg per bolus, for a maximum of three boluses. If signs of shock persist, inotropic support should be considered [25].

Close monitoring of vital signs is essential in all patients. In patients with severe dengue and shock, clinicians should aim to maintain a mean arterial pressure of 70–80 mmHg, urine output above 0.5 mL/kg/h, and a difference between systolic and diastolic blood pressure greater than 20 mmHg [26].

Since no dengue antivirals are yet approved for use in routine clinical care, management remains supportive. Risk varies according to the stage of infection and host characteristics, including age, chronic disease, and pregnancy. Appropriate management therefore requires assessment of the disease stage, risk factors, and symptom severity so that patients can be stratified by risk [27].

Dengue is characterized by febrile, critical, and recovery phases [27]. Most patients will only experience the febrile phase, followed by resolution of the fever [27]. The cornerstone of dengue management is fluid therapy, particularly during the critical phase, when fluid administration should be adjusted to the degree of plasma leakage [28]. According to the WHO, patients with dengue are classified into three management groups based on disease severity, the presence of warning signs, underlying medical conditions, and the need for clinical monitoring [27].

Group A includes patients without warning signs who tolerate oral fluids and maintain adequate urine output. These patients can generally be managed at home with appropriate hydration, symptomatic treatment, and clear instructions regarding warning signs that require immediate medical attention. Group B comprises patients with warning signs, relevant comorbidities, or social circumstances that may compromise adequate outpatient management. These patients require hospital-based monitoring and individualized fluid replacement. Group C includes patients with severe dengue who require immediate emergency treatment and, when indicated, intensive care [1].

Early identification of warning signs is essential to recognize patients at risk of progressing to severe dengue. These signs include abdominal pain or tenderness, persistent vomiting, mucosal bleeding, lethargy, hepatomegaly, and a progressive increase in hematocrit accompanied by a rapid decrease in platelet count. Severe dengue is characterized by substantial plasma leakage leading to shock or fluid accumulation with respiratory distress, severe bleeding, or severe organ involvement, including hepatic, renal, cardiac, or neurological dysfunction [29,30].

Current dengue management remains predominantly supportive, with therapeutic interventions determined by disease severity and clinical progression. Patients in Group A should receive adequate oral hydration and symptomatic treatment, including paracetamol for fever and discomfort. Aspirin and other nonsteroidal anti-inflammatory drugs should be avoided because of their potential to increase bleeding risk. Patients should also receive instructions regarding adequate fluid intake, monitoring urine output, and recognizing warning signs [24].

For patients in Group B, careful clinical monitoring and timely fluid replacement are required to prevent progression to shock. Patients with warning signs or dehydration who require intravenous therapy should receive isotonic crystalloid solutions, such as Ringer’s lactate or 0.9% saline. Fluid administration should be adjusted according to clinical response, hematocrit measurements, and hemodynamic status. Excessive fluid administration should be avoided because it may contribute to pulmonary edema, pleural effusion, or ascites, especially during the recovery phase [24].

Patients in Group C require immediate emergency management because of severe plasma leakage, circulatory shock, major bleeding, or severe organ dysfunction. Treatment primarily involves carefully monitored intravenous fluid resuscitation to restore effective circulation and maintain tissue perfusion. Isotonic crystalloids are generally used for initial fluid replacement, while additional interventions depend on the patient’s hemodynamic response and the presence of complications. Blood transfusion may be required in patients with significant hemorrhage, and persistent circulatory instability warrants further evaluation and escalation of care [1,24,30].

For the critical 48 h phase, some guidelines recommend maintenance fluids plus replacement of an estimated fluid deficit of 50 mL/kg (up to 50 kg) over that period [30]. In the critical phase of dengue fever, fluid management should maintain adequate intravascular volume while avoiding fluid overload [30]. Crystalloids are first-line therapy; if crystalloid boluses are ineffective, colloids may be considered second-line therapy [30]. Maintaining adequate perfusion and a urine output of approximately 0.5 mL/kg/h is the goal of fluid management [28]. Furthermore, during the clinical course of dengue, it is important to correct acidosis and abnormalities in blood glucose and calcium levels [30].

Patients with severe bleeding and thrombocytopenia, or those requiring emergency surgery, may receive transfusions. Blood transfusions may be used in cases of complicated dengue, such as major bleeding, severe liver involvement, or refractory acidosis. Although studies have described the benefits of steroids in dengue hemorrhagic fever, more data are needed to recommend their use. However, clinical opinion favors steroids in dengue myocarditis [30].

Some studies have suggested that N-acetylcysteine may be beneficial in patients with rising transaminase levels. Leukopenia and immune dysfunction may predispose patients to secondary bacterial sepsis, which can progress to septic shock and require appropriate antibiotic therapy [1]. Intravenous administration of anti-D globulin has been shown to improve platelet count in patients with dengue [28].

## 5. New Approaches to Prevention and Management of Dengue

### 5.1. Vaccines

#### 5.1.1. Dengvaxia

CYD-TDV is a live-attenuated vaccine that uses recombinant DNA technology to incorporate structural proteins from the four dengue virus serotypes: DENV-1 (PUO-359/TVP-1140), DENV-2 (PUO-218), DENV-3 (PaH881/88), and DENV-4 (1228/TVP-980). This vaccine is based on a chimeric platform in which the genes encoding the structural proteins (prM and E) of the four dengue virus serotypes are inserted into an attenuated viral vector, allowing the expression of dengue-specific antigens and the induction of a protective immune response against the different virus serotypes [31].

The vaccine produces antibodies directed against each viral component, although its mechanism of action is unknown [31]. Researchers evaluated its efficacy in large phase III trials in Asia and Latin America. Vaccine efficacy against symptomatic virologically confirmed dengue was 56.5% in the Asian CYD14 trial and 60.8% in the Latin American CYD15 trial, although efficacy varied substantially according to DENV serotype, age, and, importantly, baseline dengue serostatus. The major advantage of CYD-TDV is its demonstrated protection in previously infected individuals, while its principal limitation is the requirement to establish previous dengue infection before vaccination, which complicates large-scale implementation and restricts the eligible population. The CYD vaccine enhances the immune response when administered concurrently with vaccines against other pathogens. Current WHO recommendations therefore restrict its use to individuals with evidence of previous DENV infection [31]. Furthermore, the manufacturer has discontinued production of Dengvaxia because of limited global demand, further restricting its current public-health role [1,29,32,33].

#### 5.1.2. TV003/TV005 and Butantan-DV

TV003 is a quadrivalent vaccine containing three attenuated DENV components generated through a 30-nucleotide deletion (Δ30) in the 3′ untranslated region. This vaccine encodes the non-structural proteins DENV-1, DENV-3, and DENV-4, which may be essential for presenting specific DENV epitopes and providing greater protection. A 30% reduction in infectivity and viral replication in monocyte-derived dendritic cells is observed, and it elicits a strong innate immune response [34].

TV003 and TV005 are closely related single-dose, live-attenuated tetravalent dengue vaccine candidates developed by the U.S. National Institute of Allergy and Infectious Diseases (NIAID). Both vaccines contain attenuated components representing the four DENV serotypes; TV005 differs from TV003 primarily by containing a ten-fold higher dose of the DENV-2 component to achieve a more balanced tetravalent immune response. Phase I and II studies demonstrated favorable safety and immunogenicity profiles. In a randomized study conducted in dengue-endemic Bangladesh, a single dose of TV005 was well tolerated and induced seropositivity rates of 83%, 99%, 96%, and 87% against DENV-1, DENV-2, DENV-3, and DENV-4, respectively, by day 180, with immune responses observed in both previously exposed and dengue-naïve individuals. Controlled human infection studies showed complete protection against viremia and rash following experimental DENV-2 or DENV-3 challenge six months after TV005 vaccination [35,36,37,38].

Butantan-DV, a formulation derived from the TV003 platform, advanced to phase III clinical evaluation in Brazil. In the randomized, double-blind, placebo-controlled DEN-03-IB trial involving more than 16,000 participants, a single dose initially showed overall efficacy of 79.6% against symptomatic, virologically confirmed dengue after two years, with 73.6% efficacy among baseline seronegative participants and 89.2% among seropositive participants. During an extended mean follow-up of 3.7 years, overall efficacy remained 67.3%. DENV-3 and DENV-4 did not circulate sufficiently during the trial to establish field efficacy against these serotypes. Five-year follow-up has subsequently confirmed sustained protection against DENV-1 and DENV-2 [39,40,41,42].

The principal advantages of this platform are its single-dose schedule, broad age range, and efficacy irrespective of previous dengue exposure. Butantan-DV received approval in Brazil in December 2025 for individuals aged 12–59 years, becoming the first licensed single-dose dengue vaccine. However, the Brazilian Ministry of Health temporarily suspended its use in June 2026 while investigating rare, unexpected adverse events. Although its efficacy profile and single-dose regimen represent important advantages, continued pharmacovigilance remains essential [43,44,45,46,47,48].

#### 5.1.3. Qdenga or TAK-003

Perhaps one of the most encouraging studies is the application of the Qdenga preventive vaccine, also known as TAK-003, a quadrivalent vaccine suitable for inoculation from the age of 4 [35]. The vaccination regimen consists of the first dose, followed by the second dose three months later. It was created with attenuated strains of dengue virus serotypes 1, 2, 3, and 4, which facilitates an immune response through antibodies that establish an effective response to the infection. Consequently, when the person becomes infected with the virus again, their immune system will be able to recognize it and respond quickly by making more antibodies, which will stop the virus and prevent the disease from starting [35].

Unlike Dengvaxia, Qdenga offers broader population coverage, given its wider age range and the fact that prior infection is not required for its administration [35,36]. The net efficacy of this vaccine has been demonstrated in preventing dengue-associated fever in children and adolescents for 12 months following the second dose [35,36]. The analysis was based on results from over 20,000 healthy children aged 4 to 16 years who were randomized in a 2:1 ratio to receive either the TAK-003 vaccine or a placebo. All participants underwent initial serological assessments, and symptomatic dengue cases were detected throughout the study using serotype-specific reverse-transcription polymerase chain reaction (RT-PCR). Participants were enrolled in eight countries: Brazil, Colombia, the Dominican Republic, Nicaragua, Panama, Sri Lanka, and Thailand [35,36].

TAK-003 demonstrated sustained efficacy against dengue over three years. At three years, vaccine efficacy was 62% against dengue and 84% against dengue-related hospitalization. Efficacy against dengue decreased over time, whereas protection against hospitalization remained higher [36].

Furthermore, the phase III TIDES trial evaluated its efficacy and safety, enrolling more than 20,000 children and adolescents aged 4–16 years across eight dengue-endemic countries. After approximately 4.5 years of follow-up, cumulative vaccine efficacy was 61.2% against virologically confirmed dengue and 84.1% against dengue-related hospitalization. Among participants who were seronegative at baseline, efficacy was 53.5% against virologically confirmed dengue and 79.3% (95% CI, 63.5–88.2) against hospitalization [36,49].

Serious adverse events were rare, and no significant vaccine-related safety concerns were identified. Vaccination was also associated with reduced viral levels in the blood of infected individuals and sustained high levels of protective antibodies. TAK-003 therefore showed promise for preventing dengue and reducing disease severity among breakthrough infections [36].

The most common side effects of the vaccine, experienced by more than 20% of individuals, include pain and redness at the injection site, headache, muscle aches, general malaise, and weakness. Fever may occur in up to 10% of individuals. These effects, generally mild to moderate, usually disappear within a few days and are less frequent after the second dose compared to the first [35]. Administration of Qdenga is not recommended for individuals with a history of hypersensitivity to previous doses or for those with a weakened immune system due to illness, immunosuppressant medications, or HIV infection. Its use is not recommended during pregnancy or breastfeeding [35].

The vaccine provides protection against fever and hospitalization associated with dengue fever caused by any of the four serotypes of the virus. Side effects, mostly mild to moderate, tend to disappear within a few days [35]. Based on these data, the European Medicines Agency has concluded that the benefits of Qdenga outweigh its risks, thus recommending its authorization for use in the European Union [35,36].

The three vaccine platforms present distinct advantages and limitations. CYD-TDV has demonstrated efficacy in previously DENV-infected individuals but requires confirmation of prior infection because of the increased risk of severe dengue in seronegative recipients. TAK-003 does not require pre-vaccination serological screening and provides sustained protection, particularly against hospitalization; however, efficacy varies by serotype and baseline serostatus. The TV003/TV005-derived platform offers the advantage of a single-dose regimen and induces immune responses against all four serotypes. Phase III data from Butantan-DV have demonstrated sustained efficacy irrespective of baseline serostatus, although field efficacy against DENV-3 and DENV-4 remains less well established. Vaccine selection should consider previous dengue exposure, local transmission intensity and circulating serotypes, age-specific indications, dosing schedules, and evolving evidence on long-term safety and effectiveness.

### 5.2. Antiviral Drugs

#### 5.2.1. AT-752

AT-752 is an investigational oral guanosine nucleotide prodrug developed by Atea Pharmaceuticals with activity against all four DENV serotypes [39]. AT-752 targets the RNA-dependent RNA polymerase activity of DENV NS5. In a first-in-human study, it was well tolerated at single doses up to 1500 mg and multiple doses of 750 mg three times daily, with no serious adverse events; mild adverse events resolved without sequelae. Plasma exposure to the active metabolite AT-273 increased proportionally with dose, supporting its potential as an antiviral treatment for dengue [39]. Plasma exposure to the active metabolite AT-273 exceeded the antiviral threshold required to inhibit dengue virus replication, reinforcing its therapeutic potential. Furthermore, the study identified no clinically meaningful pharmacokinetic differences among Asian participants or significant effects of food, suggesting that dose adjustment based on ethnicity or dietary patterns would not be necessary [39]. In summary, these encouraging safety and pharmacokinetic findings support continued evaluation of AT-752 and informed dose selection for future clinical trials [39].

#### 5.2.2. Montelukast

The therapeutic potential of montelukast has been explored in dengue because plasma leakage is a major determinant of disease severity. Leukotrienes are lipid mediators produced primarily by mast cells that contribute to vascular leakage. During the febrile phase of dengue, leukotriene levels increase and return to baseline during recovery. This early increase has been associated with a higher risk of dengue hemorrhagic fever. As a leukotriene receptor antagonist with mast-cell-stabilizing effects, montelukast may reduce plasma leakage and the risk of hemorrhagic complications, a hypothesis supported by studies in animal models [40].

Montelukast has been evaluated in a multicenter, randomized, double-blind, placebo-controlled study conducted in Thailand. Exclusion criteria included dengue warning signs, concurrent febrile illnesses, pregnancy, and specific contraindications to montelukast. Laboratory-confirmed participants were randomly assigned in a 1:1 ratio to receive either oral montelukast (10 mg once daily) or placebo for up to 10 days or until recovery. Participants were followed for 14 days, with clinical assessments every two days and a complete blood count performed at each visit [40].

**Figure 3:**
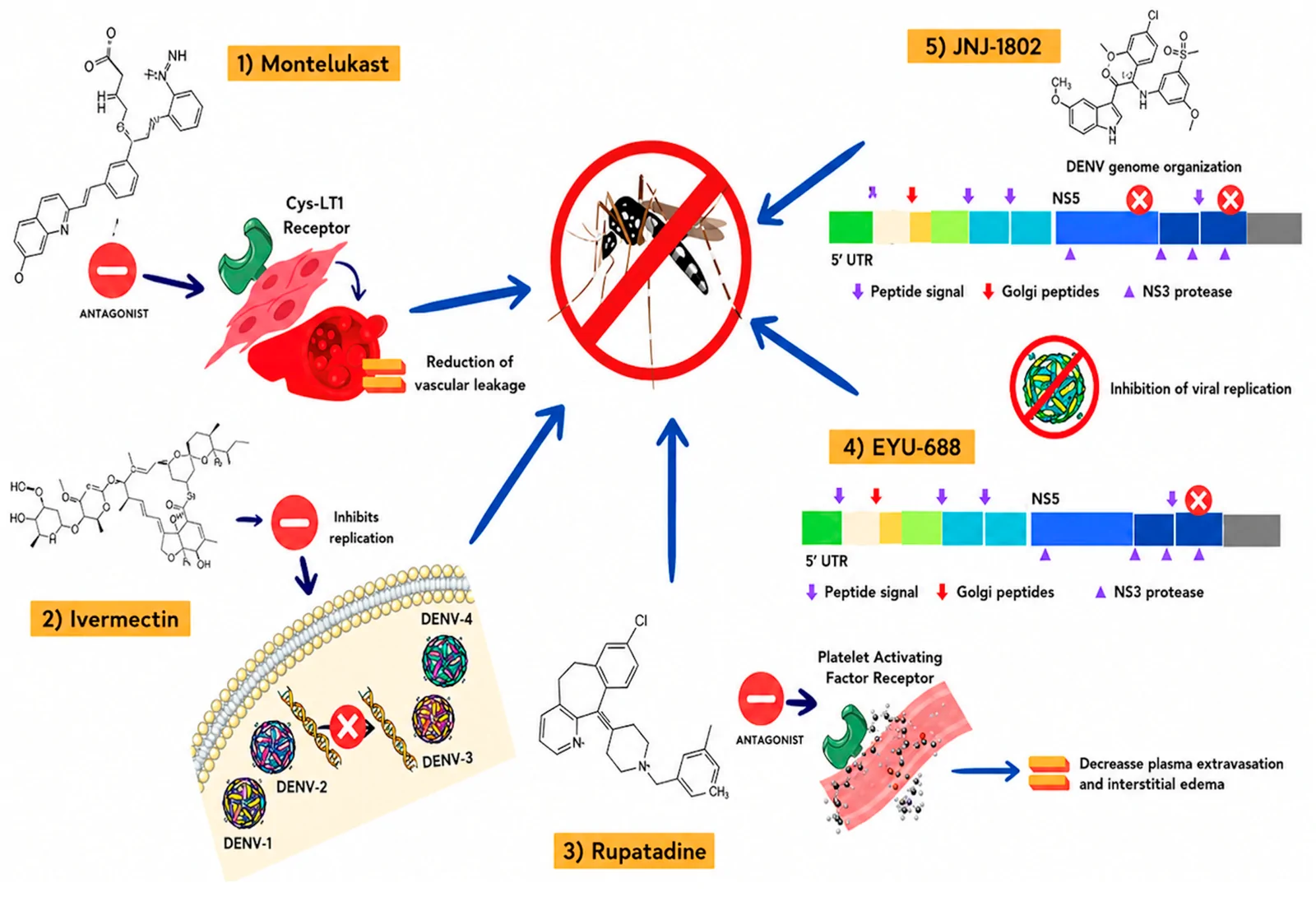
Mechanisms of action of compounds against dengue virus infection. DENV, dengue virus; Cys-LT1, cysteinyl leukotriene receptor 1; NS3, nonstructural protein 3; NS5, nonstructural protein 5; UTR, untranslated region.

Montelukast did not reduce the incidence of severe dengue or warning signs or accelerate recovery compared with placebo. However, transaminase elevations occurred less frequently in the montelukast group, suggesting a potential benefit with respect to liver enzyme elevations. These findings support further research to clarify the potential therapeutic role of montelukast in dengue and the prevention of complications (Figure 3) [40].

#### 5.2.3. Ivermectin

Ivermectin has been investigated as a potential treatment for dengue but is not approved as standard therapy for the disease. Ivermectin is an anthelmintic derived from avermectins, a class of broad-spectrum antiparasitic agents. Preclinical studies suggest that it may inhibit DENV replication; however, the evidence remains preliminary and is based largely on in vitro and animal studies. Clinical evidence of efficacy in patients with dengue is limited, and no conclusive clinical-trial evidence has established ivermectin as an effective and safe treatment for dengue in humans (Figure 3) [41].

#### 5.2.4. Rupatadine

Rupatadine is a second-generation antihistamine used to treat allergic diseases. It blocks histamine H1 receptors and also antagonizes the platelet-activating factor receptor, actions that may reduce inflammation (Table 1). Previous studies showed that rupatadine reduced endothelial dysfunction in vitro, vascular leakage in mouse models of dengue, and the extent of pleural effusions and thrombocytopenia in patients with acute dengue. A clinical trial therefore evaluated whether rupatadine could reduce the incidence of dengue hemorrhagic fever in patients with acute dengue. The study reported a trend toward a lower proportion of patients developing dengue hemorrhagic fever. Rupatadine 40 mg was reported to be safe and well tolerated in patients with acute dengue; however, its use in combination with other treatment modalities warrants further study (Figure 3) [45].

#### 5.2.5. JNJ-1802

JNJ-1802 is a small molecule that inhibits interactions between the nonstructural proteins NS3 and NS4B, thereby disrupting viral RNA synthesis and replication (Figure 3 and Table 1). The compound has demonstrated picomolar-to-nanomolar pan-serotype potency in vitro against DENV [46].

The study included a screening phase, an administration phase, and a safety follow-up period of 30 to 35 days after the last dose due to the prolonged half-life of JNJ-1802. In this clinical trial, JNJ-1802 was administered in single doses of 50, 150, 240, 400, and 1200 mg and in multiple doses of 50, 150, 400, and 560 mg. JNJ-1802 was reconstituted for oral use with PEG400 diluent to obtain 25 mL of a 10 or 160 mg/mL oral solution [46].

All adverse events across dose groups were grade 1 or 2, except for one grade 3 event (depression) in a participant who received 400 mg of JNJ-1802 for 31 days; this event was considered unrelated to the drug [46].

Adverse events considered possibly drug-related were reported in no more than one participant per group, except for diarrhea, which occurred in 71.4% of the 150 mg group, 12.5% of the 400 mg group, and 22.2% of the placebo group, and skin rash, which occurred in 12.5% of the 560 mg group and 14.3% of the 400 mg group but not in the placebo group. Diarrhea was mild, resolved within 2 to 12 days, and was attributed to the PEG400 diluent. Development of JNJ-1802 was discontinued during a phase 2 field study in October 2024 because of strategic R&D reprioritization rather than toxicity concerns. Overall, JNJ-1802 was considered safe and well tolerated [46].

#### 5.2.6. EYU688

EYU-688 is an orally administered investigational treatment for dengue that targets nonstructural protein 4B (Table 1). A randomized, parallel-group, participant- and investigator-blinded, placebo-controlled study is evaluating the efficacy, safety, and pharmacokinetics of EYU-688 in patients with dengue. The study is assessing effects on dengue viral load, time to fever resolution, and clinical signs and symptoms compared with placebo. EYU-688 is currently in phase 2 clinical development. The study began on 20 February 2024, and is expected to conclude on 30 July 2027 [49].

## 6. Advances in the Control of Aedes spp. Through Biological and Genetic Approaches as Alternatives to Chemical Control

### 6.1. Sterile Insect Technique by Irradiation

The sterile insect technique (SIT) is an environmentally oriented vector-control strategy based on the mass production, sterilization, and release of male mosquitoes. Males are sterilized by exposure to ionizing radiation, usually during the pupal stage, while retaining their ability to mate with wild females. These matings produce nonviable eggs, progressively reducing the target mosquito population [50,51,52,53].

Field studies have demonstrated substantial mosquito suppression using this approach. In one trial with *Aedes aegypti*, weekly releases of approximately 9000 irradiated males per hectare for 24 weeks reduced egg hatching by approximately 86%, although mosquito populations rebounded after releases were discontinued [51]. These findings indicate that the effectiveness of SIT depends on sustained releases, male competitiveness, mosquito immigration, and adequate management of breeding sites [50,51,52,53,54,55,56,57].

Combining irradiation with the incompatible insect technique mediated by *Wolbachia* may further improve population suppression. Zheng et al. reported near elimination of two field populations of *Aedes albopictus* using an incompatible insect technique (IIT)-SIT strategy, in which Wolbachia-induced cytoplasmic incompatibility was combined with irradiation to prevent reproduction by accidentally released females [50].

### 6.2. RIDL/RISL Genetic Lethality Systems

Release of Insects carrying a Dominant Lethal gene (RIDL), also referred to as Release of Insects carrying a Self-Limiting gene (RISL), is a genetic population-suppression strategy in which modified male mosquitoes carry a lethal genetic construct that causes their offspring to die before reaching maturity [58,59,60,61,62]. Because the modification is self-limiting, it gradually disappears from the population when releases cease [58,60].

Field trials with the OX513A strain of *Aedes aegypti* have demonstrated substantial population suppression. In Brazil, sustained releases resulted in a 95% reduction in the local adult mosquito population and a 78–81% reduction based on ovitrap measurements [59]. Similar suppression was observed in the Cayman Islands. However, effectiveness depends on maintaining sufficient numbers of modified males, their mating competitiveness, and limiting immigration of wild mosquitoes from untreated areas [59].

RIDL/RISL systems are species-specific and offer greater control over the persistence of the genetic modification compared with self-sustaining approaches [58,59,60,61,62]. Nevertheless, local mosquito density, migration, seasonal variation, release capacity, public acceptance, and long-term sustainability should be considered before implementation [60,61,62].

### 6.3. Female-Specific Lethality Strategies (Female-Killing)

Female-specific lethality strategies use genetic constructs whose lethal effect is restricted to female offspring. Modified males mate with wild females and transmit the construct to their progeny; female descendants die, whereas male descendants may continue transmitting the construct for a limited number of generations, thereby contributing to population suppression [63].

Mathematical modeling by Phuc et al. suggested that late-acting lethality may be more efficient than early embryonic lethality in *Aedes aegypti*. The estimated release ratio required for population elimination was 27–54% greater for early-acting than for late-acting lethality, depending on the strength of density-dependent regulation [63]. Thus, the timing of lethality represents an important determinant of the efficiency of this strategy.

### 6.4. Biological Control Using Wolbachia

Biological control using *Wolbachia* aims primarily to reduce the ability of *Aedes aegypti* populations to transmit arboviruses rather than eliminate the mosquito population. *Wolbachia* is a maternally inherited intracellular bacterium that spreads within mosquito populations via cytoplasmic incompatibility, allowing selected strains to become established and persist locally [64,65,66,67,68,69,70,71,72].

In a cluster-randomized trial conducted in Yogyakarta, Indonesia, deployment of wMel-infected *Aedes aegypti* reduced virologically confirmed symptomatic dengue by 77.1% and dengue-related hospitalizations by 86.2%, with protection observed against all four dengue virus serotypes [72]. The reduction in transmission has been associated with interference in viral replication and dissemination within the mosquito [64,65].

Environmental conditions can influence the stability of *Wolbachia* infections. High temperatures may affect bacterial density, maternal transmission, and cytoplasmic incompatibility. The wAlbB strain has therefore been investigated as an alternative for warmer environments. In dengue-endemic areas of Kuala Lumpur, Malaysia, wAlbB-infected *Aedes aegypti* became established at high frequency and was associated with reductions in reported dengue incidence, although additional releases were required in some locations [65].

Municipality-wide Wolbachia release programs conducted between 2017 and 2024 in Bello, Medellín, and Itagüí, Colombia, covering more than 3.5 million people, were associated with substantial and sustained reductions in dengue incidence. Reductions of 91.9% and 92.1% were reported in Bello and Itagüí, respectively, with protection remaining evident even during the major national dengue outbreak of 2024. These findings support the potential of Wolbachia-based interventions as a complementary strategy for dengue prevention and vector control in endemic settings.

The World Mosquito Program initiative in Colombia is part of a global non-profit effort aimed at protecting communities from mosquito-borne diseases. In Colombia, more than 25 million people are considered at risk of dengue infection, representing more than half of the country’s population. The country has experienced several large-scale dengue outbreaks in recent years, while Zika virus infections also increased markedly following the global Zika outbreak that began in 2015. The World Mosquito Program has implemented its self-sustaining Wolbachia method in Colombia to reduce the ability of Aedes mosquitoes to transmit dengue, Zika, and chikungunya viruses [73,74]. Overall, Wolbachia-based biological control has demonstrated substantial potential for reducing dengue transmission. wMel currently has strong evidence from randomized trials, whereas wAlbB may provide advantages under very warm environmental conditions [64,65,73].

## 7. Perspectives and Conclusions

Given the limited therapeutic options and the high incidence of dengue currently, it is important to create other epidemiological surveillance systems, including integrated entomology, to address the incidence and implementation of intersectoral actions, provide care for patients both within and outside the healthcare system, report cases, and conduct a critical analysis of the use and function of insecticides. Formal health training should be provided for professionals and individuals working in the medical and social sciences, thereby further educating the community. It is also important that local and national authorities implement effective control and monitoring strategies. These measures can help reduce the spread of dengue and the number of cases.

The urgent need to develop and improve therapeutic strategies against dengue is driven by its persistent global impact, highlighting the inadequacy of existing therapeutic approaches to address this infection. Ongoing research and collaboration among scientists, medical institutions, and public health agencies are essential to drive the development and implementation of new dengue therapeutic strategies, with the goal of improving clinical outcomes and reducing the morbidity and mortality associated with this disease. While much remains to be done, recent advances in understanding and treating dengue provide cause for optimism, emphasizing the necessity of continuing to explore new therapeutic avenues and promoting research and innovation in this critical area of global public health.

## Figures and Tables

**Table 1:** Mechanisms of action, adverse effects, and outcomes of drugs evaluated in clinical trials for dengue treatment.

Medicine	Clinical Trial	Mechanism of Action	Adverse Effects	Results	Conclusions
Montelukast	NCT04673422	A leukotriene receptor antagonist that reduces vascular leakage and may therefore reduce the development of dengue hemorrhagic fever.	No adverse effects were reported.	No efficacy was demonstrated in reducing dengue incidence. However, transaminase elevations occurred less frequently than in the placebo group.	Treatment with 10 mg of montelukast for 10 days did not decrease the incidence of dengue hemorrhagic fever. Further research is needed to assess the effect on transaminases.
Ivermectin	NCT02045069	A semisynthetic oral anthelmintic that inhibits replication of all four DENV serotypes in vitro.	Reported adverse effects were unrelated to ivermectin and reflected signs and symptoms of dengue.	An oral dose of 400 µg/kg was safe and accelerated the elimination of the NS1 antigen in patients with dengue but did not reduce viremia levels.	NS1 elimination was achieved without significant clinical benefit; therefore, early administration of higher doses has been suggested.
Rupatadine	SLCTR/2017/024	An antagonist of the platelet-activating factor receptor that reduces plasma extravasation and tissue edema.	No adverse effects were reported.	Rupatadine was safe and well tolerated and reduced the development of dengue hemorrhagic fever.	Rupatadine decreased the development of dengue hemorrhagic fever. Its usefulness in combination with other treatments should be explored.
JNJ-1802	https://doi.org/10.1093/cid/ciad284	It inhibits the formation of the non-structural proteins NS3 and NS4B, thereby preventing viral replication.	Diarrhea attributed to the PEG400 diluent and skin rashes resolved after treatment cessation.	JNJ-1802 proved to be safe and well tolerated in healthy participants.	Pharmacokinetic and safety results should be confirmed in patients with dengue.
EYU-688	NCT06006559	Targets nonstructural protein 4B (NS4B)	-	-	-

## Data Availability

No new data were created or analyzed in this study. Data sharing is not applicable to this article.
